# Post-transcriptional regulation of mRNA stability in pancreatic ductal adenocarcinoma

**DOI:** 10.3389/fimmu.2026.1861092

**Published:** 2026-06-03

**Authors:** Heyang Wang, Pan Liu, Juntaro Yamasaki, Tatsuhiko Harada, Tatsuya Sakaguchi, Tetsuya Takimoto, Hideyuki Saya, Osamu Nagano, Kazuhiro Fukumura

**Affiliations:** 1 Division of Gene Regulation, Oncology Innovation Center, Fujita Health University, Toyoake, Japan; 2 Department of Pharmacy, Women’s Hospital, Zhejiang University School of Medicine, Hangzhou, Zhejiang, China; 3 Department of Advanced Robotic and Endoscopic Surgery, Fujita Health University, Toyoake, Japan; 4 Department of Dentistry and Oral Surgery, Keio University School of Medicine, Tokyo, Japan

**Keywords:** epithelial–mesenchymal transition (EMT), m6A epitranscriptomics, metabolic reprogramming, mRNA stability, pancreatic ductal adenocarcinoma (PDAC), phenotypic plasticity, post-transcriptional regulation, RNA-binding proteins (RBPs)

## Abstract

Pancreatic ductal adenocarcinoma (PDAC) remains one of the most lethal malignancies and is characterized by pronounced phenotypic plasticity, metabolic adaptation, and therapeutic resistance within a dense and desmoplastic tumor microenvironment. Although transcriptional deregulation has been extensively investigated, post-transcriptional regulation, particularly the control of mRNA stability, has emerged as a critical and previously underexplored contributor to PDAC progression. RNA-binding proteins (RBPs), together with cis-regulatory RNA elements and epitranscriptomic modifications such as N6-methyladenosine (m6A), form interconnected regulatory networks that dynamically modulate mRNA turnover and thereby shape protein output in response to microenvironmental stress. By selectively stabilizing transcripts encoding epithelial–mesenchymal transition (EMT) regulators, metabolic enzymes, and stress-response factors, these networks promote reversible, non-genetic adaptation without requiring permanent genetic alterations. This regulatory flexibility supports invasion, therapeutic tolerance, and intratumoral heterogeneity under hypovascular and nutrient-limited conditions. Recent advances further suggest that targeting mRNA stability through small molecules and RNA-directed strategies may provide new therapeutic opportunities in PDAC. In this review, we summarize current insights into post-transcriptional mechanisms regulating mRNA stability in PDAC, highlight key knowledge gaps, and discuss their potential translational implications.

## 1 Introduction

Pancreatic ductal adenocarcinoma (PDAC) remains one of the most lethal solid malignancies, with a 5-year overall survival rate of approximately 10%, largely due to late diagnosis, early metastatic spread, and profound resistance to conventional therapies (1). In addition, PDAC is characterized by dense desmoplastic stroma, severe hypovascularity, and an immunosuppressive microenvironment, all of which further limit therapeutic efficacy (2). Although combination chemotherapy regimens such as FOLFIRINOX and gemcitabine plus nab-paclitaxel have modestly improved outcomes, durable clinical benefit remains rare, underscoring the need for deeper mechanistic insights into PDAC biology (3, 4). Recurrent oncogenic alterations, including mutations in KRAS, TP53, CDKN2A, and SMAD4, are well established as initiating and driving events in PDAC (5). However, these genomic lesions alone do not fully explain the pronounced phenotypic heterogeneity, transcriptional plasticity, and adaptive capacity characteristic of PDAC tumors (6). A recent integrative analysis of The Cancer Genome Atlas (TCGA) and the International Cancer Genome Consortium (ICGC) datasets revealed m6A-related gene expression signatures that stratify PDAC into distinct prognostic subgroups. Notably, these subgroups differed significantly in KRAS mutation status, suggesting a potential link between oncogenic KRAS signaling and m6A-dependent post-transcriptional regulation in PDAC (7). Increasing evidence from transcriptomic and epigenomic studies indicates that regulatory layers operating downstream of transcription play essential roles in shaping malignant gene-expression programs, particularly under metabolic stress, hypoxia, and therapeutic pressures characteristic of the PDAC microenvironment (8).

Among post-transcriptional regulatory processes, control of mRNA stability has emerged as a critical determinant of transcript abundance and protein output. Unlike transcriptional regulation, modulation of mRNA stability enables rapid and reversible adaptation to environmental cues without requiring extensive chromatin remodeling. Dysregulated mRNA stability has been implicated in oncogenic signaling, stress tolerance, and therapy resistance across multiple cancer types, highlighting its functional relevance in tumor progression. In PDAC, this mode of regulation may be especially important for understanding how tumor cells adapt to dynamic microenvironmental stress and therapeutic pressure.

RNA-binding proteins (RBPs) serve as central effectors of mRNA stability control by interacting with specific sequence and structural elements within target transcripts. In PDAC, aberrant expression or activity of RBPs, including IGF2BP family proteins, HuR, and YTH-domain readers, has been linked to sustained oncogenic signaling, metabolic reprogramming, and epithelial–mesenchymal plasticity (9–11). Despite growing interest in RBPs in pancreatic cancer, most studies have focused on individual proteins or discrete regulatory events, leaving the broader mechanistic principles governing mRNA stability insufficiently defined (12). In this review, we discuss how dysregulated post-transcriptional networks control mRNA stability in PDAC and contribute to tumor plasticity, progression, and therapeutic resistance. We synthesize recent advances in RBP–3′UTR interactions, RNA modification-dependent regulation, and post-transcriptional network dynamics, and we highlight key conceptual and translational questions in this emerging field.

## 2 General mechanisms of mRNA stability control in PDAC

### 2.1 RBP–3′UTR interactions and cis-regulatory elements

RBPs determine transcript fate by recognizing specific cis-regulatory elements within target mRNAs and integrating cellular context with RNA structural and chemical features to modulate stability. The 3′ untranslated region (3′UTR) serves as the principal platform for this regulation, containing binding sites for RBPs and associated cofactors that coordinate transcript decay and translation. Among these elements, AU-rich elements (AREs) are extensively characterized and are enriched in transcripts encoding growth factors, stress mediators, and inflammatory regulators, allowing dynamic regulation of mRNA half-life through RBP interaction. Beyond primary sequence, RNA secondary and higher-order structures within 3′UTRs strongly influence RBP recognition. Structural motifs such as stem-loops or bulges can expose or occlude binding sites, enabling transcript-selective regulation and increasing regulatory complexity in cancer cells (13). Alternative polyadenylation (APA) further expands 3′UTR-dependent control by generating isoforms with variable 3′UTR lengths. Shortened 3′UTRs may escape repressive RBPs, whereas extended regions can introduce additional destabilizing elements. In cancer, APA-mediated 3′UTR shortening has been associated with increased transcript stability and enhanced protein output of oncogenic isoforms (14, 15).

**Figure 1:**
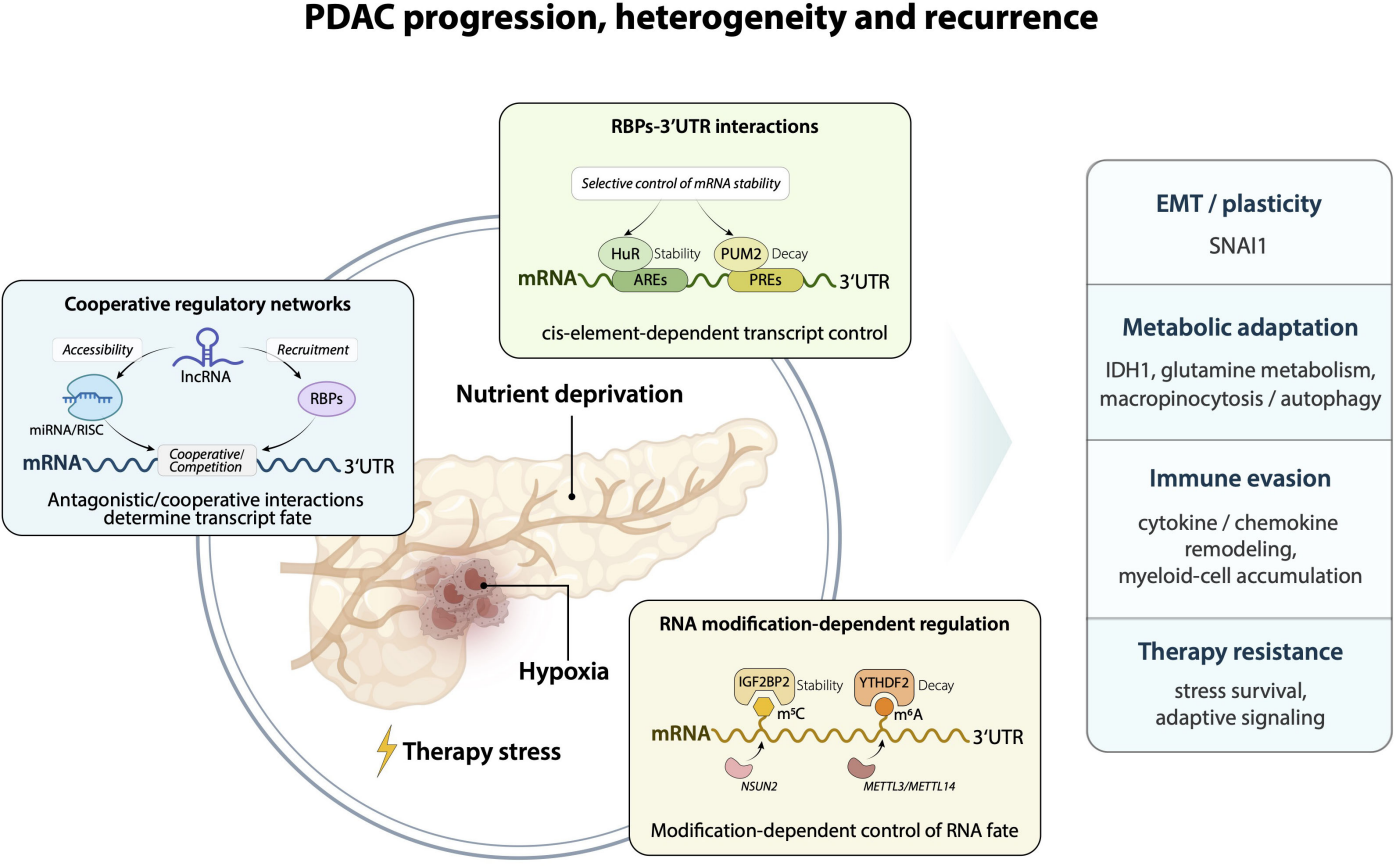
Post-transcriptional mechanisms regulating mRNA stability in PDAC. The figure summarizes major mechanisms of mRNA stability control in pancreatic ductal adenocarcinoma (PDAC), including RBP–3′UTR interactions, RNA modification-dependent regulation, and cooperative networks involving RBPs, miRNAs, and lncRNAs. These pathways are activated or remodeled under nutrient deprivation, hypoxia, and therapy stress, and contribute to EMT/plasticity, metabolic adaptation, immune evasion, and therapy resistance, thereby promoting PDAC progression, heterogeneity, and recurrence.

In addition to these structural and sequence-dependent mechanisms, PDAC-relevant RBPs can also directly regulate transcript stability. For example, PUM2 has been shown to promote chemoresistance by stabilizing focal-adhesion-related transcripts through 3′UTR-dependent mechanisms, illustrating how RBP-mediated mRNA stabilization can drive malignant phenotypes in pancreatic cancer (16). Collectively, these mechanisms show how cis-elements, RNA structure, and APA dynamics shape mRNA stability in PDAC through RBP–3′UTR interactions (**Figure 1**).

### 2.2 RNA modification–dependent regulation of mRNA stability

Chemical RNA modifications add an additional regulatory layer beyond primary sequence. Among them, N6-methyladenosine (m6A) is the most prevalent and dynamically reversible modification associated with transcript turnover (17). m6A regulates mRNA fate in a context-dependent manner that is shaped by cellular state and the composition of post-transcriptional regulatory networks. m6A is deposited by the METTL3–METTL14 methyltransferase complex, in which METTL3 functions as the catalytic subunit and METTL14 supports substrate recognition, and is enriched near stop codons and within 3′UTRs, spatially overlapping with classical post-transcriptional regulatory regions (17, 18). This localization enables m6A to influence RNA structure and recruit selective RBPs. Functional outcomes depend largely on reader proteins: YTHDF2 promotes decay through deadenylation and exonucleolytic pathways, whereas IGF2BP family members stabilize m6A-modified transcripts and enhance their persistence (19, 20).

In PDAC, dysregulation of m6A machinery can affect transcript stability in a target-specific manner. For example, METTL3 has been shown to promote pancreatic cancer growth and metastasis by enhancing the m6A-dependent stability of transcription factor E2F5 mRNA (21). These findings indicate that epitranscriptomic regulation broadens the post-transcriptional flexibility of gene-expression programs in PDAC (**Figure 1**).

### 2.3 Cooperative and competitive interactions among post-transcriptional regulators

mRNA stability is often shaped by the combined effects of cooperative and competitive interactions among RBPs, miRNAs, lncRNAs and RNA-modification readers, rather than by any single regulator alone. These factors frequently converge on shared cis-elements within the same transcript, forming dynamic regulatory assemblies that vary with cellular context (22). In addition to directly affecting transcript stability, lncRNAs can function as molecular scaffolds or decoys that modulate the recruitment, accessibility, or activity of RBPs and miRNA-associated complexes, thereby reshaping post-transcriptional outputs in a context-dependent manner. Cooperative interactions can enhance either transcript stabilization or decay. For example, binding of one RBP may remodel local RNA structure and alter the accessibility of nearby miRNA target sites or RNA-binding surfaces, thereby shifting the stability of the target transcript (23).

In contrast, competitive interactions commonly arise when RBPs and miRNAs target overlapping or adjacent sites within the 3′UTR. A well-established example is HuR, which can attenuate miRNA-mediated repression by promoting dissociation of miRISC from target transcripts, thereby shifting them toward a more stable state (23). In PDAC, these regulatory interactions are integrated into disease-relevant circuits, including pathways that regulate EMT through changes in Snail and SNAI1 mRNA stability (24, 25). Taken together, these examples indicate that altered mRNA stability in PDAC is not controlled by isolated regulators but instead emerges from context-dependent cooperation and competition among RBPs, miRNAs, lncRNAs and RNA-modification pathways (**Figure 1**).

## 3 Functional consequences of altered mRNA stability in PDAC

### 3.1 Plasticity, EMT, and stress adaptation

Phenotypic plasticity enables PDAC cells to reversibly shift between epithelial and mesenchymal states, thereby promoting invasion, dissemination, and survival under hostile microenvironmental conditions such as hypoxia, nutrient deprivation, and therapeutic stress. Although epithelial–mesenchymal transition (EMT) is initiated by transcriptional programs, maintenance of mesenchymal phenotypes also depends on post-transcriptional regulation, particularly through control of mRNA stability. Under hypoxic or treatment-induced stress, RNA regulators can reshape the stability of EMT-associated transcripts, enabling rapid and reversible phenotypic changes without requiring permanent genetic alteration.

For example, HuR stabilizes Snail mRNA, thereby prolonging its half-life and sustaining Snail protein expression, which in turn promotes EMT, metastasis, and stem-like features in cancer (26, 27). In this context, post-transcriptional control acts by preventing rapid degradation of an EMT-promoting transcript, allowing tumor cells to maintain a mesenchymal and invasive state. By contrast, the lncRNA GATA6-AS1 suppresses hypoxia-induced EMT by reducing SNAI1 mRNA stability through the m6A demethylase FTO (25). This destabilization likely shortens the persistence of SNAI1 transcripts and limits the accumulation of SNAI1 protein under hypoxic stress. Together, these examples suggest that EMT in PDAC is regulated not only at the transcriptional level but also through dynamic control of mRNA turnover, enabling rapid and reversible phenotypic adaptation to microenvironmental stress. Tumor subpopulations frequently adopt hybrid epithelial/mesenchymal states associated with metastatic potential and drug tolerance. Consistent with this view, EMT plasticity in PDAC appears to be more closely linked to chemoresistance and adaptive survival than to metastasis itself (24, 25). Taken together, altered mRNA stability represents a key mechanism linking EMT plasticity to therapeutic resistance in PDAC. Given the profound hypoxic stress characteristic of PDAC, m6A-dependent regulation of mRNA stability may play a particularly important role in sustaining these adaptive gene-expression programs (28).

### 3.2 Metabolic adaptation and survival signaling

To survive chronic nutrient limitation, PDAC undergoes extensive metabolic reprogramming that supports biosynthesis, redox balance, and pro-survival signaling. A key feature of this metabolic plasticity is post-transcriptional control of mRNA stability, which can dynamically regulate transcripts encoding metabolic enzymes, nutrient transporters, and redox regulators. Specific RNA regulators also contribute directly to metabolic adaptation in PDAC.

One example is IGF2BP2, an m6A reader that binds and stabilizes transcripts involved in glutamine utilization, most notably the glutamine transporter pathway centered on SLC1A5. By sustaining SLC1A5 expression, IGF2BP2 enhances glutamine uptake and activates mTORC1-dependent anabolic signaling, thereby supporting the metabolic demands of pancreatic cancer cells and promoting tumor progression (29). Because glutamine is an important nutrient for tumor growth, this pathway links RNA regulation to a metabolic program that helps PDAC cells survive under metabolic stress. Another example is ALKBH5, an m6A demethylase that functions under hypoxic stress. Under low-oxygen conditions, ALKBH5 reduces m6A modification on HDAC4 mRNA, thereby increasing its stability and expression. Elevated HDAC4 then enhances HIF1α protein stability, while HIF1α in turn promotes ALKBH5 transcription, forming a positive feedback loop that supports glycolysis and cell migration in pancreatic cancer cells (28). Together, these findings show that RNA regulators help PDAC cells adapt to metabolic stress by sustaining nutrient use and reshaping energy-producing pathways.

### 3.3 Immune-related effects and microenvironmental remodeling

Dysregulated mRNA stability in PDAC can also influence tumor–immune interactions by reshaping the abundance of cytokine, chemokine, and inflammation-associated transcripts. One example is IGF2BP2: in PDAC, this m6A reader promotes immune evasion by stabilizing transcripts involved in SGMS2-dependent sphingomyelin metabolism. By enhancing the persistence of these mRNAs, IGF2BP2 sustains sphingomyelin synthesis and thereby supports membrane lipid-raft formation, which facilitates PD-L1 localization at the cell surface. Through this mechanism, altered mRNA stability is linked directly to reduced effector immune-cell infiltration and the establishment of an immunosuppressive tumor microenvironment (30).

Altered RNA stability may also affect immune-related pathways indirectly through stromal cells. For instance, PDAC-derived exosomal tRF-GluCTC-0005, a tRNA-derived RNA fragment, increases WDR1 mRNA stability in hepatic stellate cells and is associated with increased secretion of IL-6, IL-10, and IL-1α (31). These cytokines, together with enhanced MDSC recruitment, help establish an inflammatory and immunosuppressive microenvironment, thereby promoting an immunosuppressive pre-metastatic niche. These findings suggest that altered mRNA stability in PDAC can influence immune-related pathways through downstream remodeling of tumor- and stroma-derived signaling programs.

## 4 Therapeutic potential of targeting RNA stability control in PDAC

### 4.1 Examples of preclinical studies and emerging therapeutic strategies targeting RNA stability control in PDAC

Several studies support the idea that RNA stability control is therapeutically targetable in PDAC, even though no such therapy has yet been established clinically. Current approaches can be broadly divided into two categories: direct inhibition of upstream RNA regulatory factors and transcript-directed RNA therapeutics aimed at disease-driving downstream transcripts (**Table 1**). However, the level of evidence differs across targets, with some strategies remaining at the proof-of-principle stage and others already entering early clinical development.

**Table 1:** Representative RNA-binding proteins (RBPs) and epitranscriptomic regulators implicated in the control of mRNA stability in pancreatic ductal adenocarcinoma (PDAC).

Regulator	Category	Main target(s)/pathway	Effect on mRNA stability or RNA fate	Functional consequence in PDAC	References
HuR (ELAVL1)	RBP	IDH1, SNAI1, stress-response transcripts, microenvironment-associated signaling factors	Stabilizes target mRNAs through 3′UTR binding and shapes stromal/immune signaling	Promotes metabolic adaptation, EMT, metastasis, stress survival, and tumor microenvironment remodeling	(10, 24)
IGF2BP2	m6A reader/ RBP	SLC1A5–mTORC1 axis, SGMS2-dependent sphingomyelin metabolism, immune-evasion pathways	Stabilizes m6A-modified transcripts and enhances transcript persistence	Promotes glutamine metabolism, tumor progression, immune evasion, and adaptive survival	(29)
YTHDF2	m6A reader	m6A-modified transcripts; RNA programs linked to EMT and proliferation	Promotes transcript decay in a context-dependent manner	Contributes to transcript turnover associated with EMT/proliferation-related phenotypes in PDAC	(11)
METTL3	m6A writer	E2F5, glycolysis-related pathways	Increases m6A deposition and reshapes transcript stability	Promotes PDAC growth, metastasis, and metabolic adaptation	(21)
GATA6-AS1–FTO axis	lncRNA-associated m6A regulatory axis	SNAI1, hypoxia-responsive transcripts	Modulates m6A-dependent RNA fate and promotes destabilization of SNAI1 mRNA	Suppresses hypoxia-induced EMT and limits adaptive plasticity	(25)
PUM2	RBP	Focal-adhesion-related transcripts, EGR1-associated pathway	Stabilizes target transcripts through 3′UTR-dependent mechanisms	Promotes chemoresistance and malignant adaptation	(16)

The table summarizes major targets or pathways, principal effects on RNA fate, and functional consequences reported in PDAC-related contexts.

One prominent example is METTL3, an m6A writer for which small-molecule inhibitors have been developed. These include STM2457, which has shown activity in preclinical studies, and the related inhibitor STC-15, which has entered phase 1 clinical testing in advanced solid tumors (ClinicalTrials.gov: NCT05584111). Although these agents have not been developed specifically for PDAC, they suggest that the RNA methylation machinery may be pharmacologically tractable and provide a rationale for exploring whether this strategy could be extended to pancreatic cancer. Given that METTL3 has been implicated in PDAC growth, metastasis, and regulation of RNA stability, this class of inhibitors illustrates the potential therapeutic potential of targeting epitranscriptomic regulators in PDAC.

Another example is IGF2BP2, an m6A reader that functions as a stabilizer of disease-promoting transcripts in PDAC. Direct pharmacological targeting of IGF2BP2 is also beginning to emerge. The small-molecule compound CWI1–2 has been reported as an IGF2BP2 inhibitor in preclinical studies, although its activity has so far been characterized mainly outside PDAC (32). Thus, the current evidence is more limited than for METTL3, but it nevertheless provides proof of principle that IGF2BP2 itself may be druggable. Because IGF2BP2 supports glutamine-dependent metabolic adaptation and immune evasion in PDAC, its inhibition could in principle disrupt multiple adaptive pathways at once.

Transcript-directed approaches also remain relevant. Antisense oligonucleotides (ASOs) and related RNA-based strategies may be particularly useful when disease-driving transcripts can be clearly defined as key outputs of RNA stability pathways. In pancreatic cancer cells, antisense targeting of BCL2L1/Bcl-xL reduced Bcl-xL expression, induced apoptosis, and increased gemcitabine sensitivity, providing a practical preclinical example of RNA-level therapeutic intervention in this disease (33). Although this strategy targets a downstream transcript rather than an upstream RNA stability regulator, it illustrates an important complementary therapeutic concept: pathogenic RNA outputs of post-transcriptional control may themselves be actionable.

Together, these studies suggest that PDAC may be targetable at multiple levels of RNA stability control, including upstream regulators, downstream pathways, and pathogenic transcripts themselves. At present, METTL3 represents the most advanced example of direct pharmacological intervention, whereas IGF2BP2 and transcript-directed RNA therapeutics highlight additional avenues that may become increasingly relevant as target validation and drug development progress.

### 4.2 Current challenges and future directions

Despite this promise, major challenges remain. A central difficulty is that regulators of RNA stability often control broad RNA networks rather than single transcripts. Factors such as METTL3 and IGF2BP2 influence multiple pathways involved in tumor growth, metabolism, and immune escape, raising the possibility that therapeutic intervention could produce on-target toxicity or trigger compensatory responses through parallel mechanisms (34).

A second challenge is context dependence. In PDAC, the importance of a given RNA regulator may change across hypoxia, nutrient limitation, stromal interaction, and treatment exposure. As a result, target dependence may not be constant across all tumor cells or disease stages, which complicates biomarker development and makes it unlikely that expression alone will be sufficient to predict therapeutic response (35–37). More informative biomarkers may need to capture cell state, regulator activity, or transcript-level outputs of RNA stability control.

A third challenge is drug delivery and selectivity. Even if disease-relevant transcripts or regulators are identified, PDAC remains a difficult therapeutic setting because of its dense stroma, poor perfusion, and marked inter- and intratumoral heterogeneity. These features may limit uptake of RNA therapeutics and reduce the consistency of target inhibition across the tumor. Future progress will therefore depend on improved experimental models, better state-resolved analysis of RNA stability programs, and clearer identification of disease-relevant and therapeutically accessible dependencies. Ultimately, the translational value of this field will depend not only on showing that RNA stability control is biologically important in PDAC, but also on defining where it can be targeted with sufficient specificity and therapeutic benefit.

## 5 Conclusions and perspectives

Control of mRNA stability has emerged as an important layer of gene regulation in pancreatic ductal adenocarcinoma (PDAC). As discussed in this review, RNA-binding proteins, epitranscriptomic modifications, and cooperative post-transcriptional interactions shape the fate of transcripts involved in cellular plasticity, metabolic adaptation, and tumor–microenvironment crosstalk. In this sense, altered mRNA stability is not simply a downstream consequence of oncogenic signaling, but an active mechanism that helps PDAC cells survive and adapt under hypoxia, nutrient limitation, and treatment pressure.

Current evidence indicates that dysregulated mRNA stability contributes to key features of PDAC biology, including adaptive plasticity, metabolic reprogramming, and immune evasion. From a translational perspective, this regulatory layer is increasingly relevant, as representative RNA regulatory factors such as METTL3 and IGF2BP2, together with transcript-directed approaches such as antisense oligonucleotides, suggest that PDAC may be targetable not only through oncogenic signaling pathways but also through adaptive RNA regulatory programs.

At the same time, major challenges remain. RNA stability regulators often act through broad and context-dependent networks, complicating target selection, biomarker development, and prediction of therapeutic response. In addition, the dense stroma, poor perfusion, and marked heterogeneity of PDAC continue to limit effective drug delivery and consistent target inhibition. Overall, further progress will depend on improved experimental models, better state-resolved analysis of RNA stability programs, and clearer identification of therapeutically accessible dependencies. A deeper understanding of these pathways may ultimately help define new strategies to overcome plasticity, metabolic adaptation, and treatment resistance in PDAC.
